# Distinct ecological fitness factors coordinated by a conserved *Escherichia coli* regulator during systemic bloodstream infection

**DOI:** 10.1073/pnas.2212175120

**Published:** 2022-12-27

**Authors:** Nicky O’Boyle, Gillian R. Douce, Gillian Farrell, Nicholas J. W. Rattray, Mark A. Schembri, Andrew J. Roe, James P. R. Connolly

**Affiliations:** ^a^School of Microbiology, University College Cork, National University of Ireland, Cork T12 K8AF, Ireland; ^b^Institute of Infection, Immunity and Inflammation, University of Glasgow, Glasgow G12 8TA, United Kingdom; ^c^Strathclyde Institute of Pharmacy and Biomedical Sciences, University of Strathclyde, Glasgow G4 0RE, United Kingdom; ^d^School of Chemistry and Molecular Biosciences, University of Queensland, Brisbane, QLD 4072, Australia; ^e^Newcastle University Biosciences Institute, Newcastle University, Newcastle-upon-Tyne NE2 4HH, United Kingdom

**Keywords:** *Escherichia coli*, regulation, bloodstream, fimbriae, tryptophan

## Abstract

The ability of bacterial pathogens to adapt to host niches is driven by the carriage and regulation of genes that benefit pathogenic lifestyles. Genes that encode virulence or fitness-enhancing factors must be regulated in response to changing host environments to allow rapid response to challenges presented by the host. Furthermore, this process can be controlled by preexisting transcription factors (TFs) that acquire new roles in tailoring regulatory networks, specifically in pathogens. However, the mechanisms underlying this process are poorly understood. The highly conserved *Escherichia coli* TF YhaJ exhibits distinct genome-binding dynamics and transcriptome control in pathotypes that occupy different host niches, such as uropathogenic *E. coli* (UPEC). Here, we report that this important regulator is required for UPEC systemic survival during murine bloodstream infection (BSI). This advantage is gained through the coordinated regulation of a small regulon comprised of both virulence and metabolic genes. YhaJ coordinates activation of both Type 1 and F1C fimbriae, as well as biosynthesis of the amino acid tryptophan, by both direct and indirect mechanisms. Deletion of *yhaJ* or the individual genes under its control leads to attenuated survival during BSI. Furthermore, all three systems are up-regulated in response to signals derived from serum or systemic host tissue, but not urine, suggesting a niche-specific regulatory trigger that enhances UPEC fitness via pleiotropic mechanisms. Collectively, our results identify YhaJ as a pathotype-specific regulatory aide, enhancing the expression of key genes that are collectively required for UPEC bloodstream pathogenesis.

## Significance

Bacterial pathogens often acquire virulence genes that drive infection. However, preexisting genes related to metabolism can also be tailored to enhance pathogen fitness within the host by adapting their transcriptional control. Here, we describe a unique mechanism by which uropathogenic *Escherichia coli* (UPEC)*,* the most common cause of urinary tract and bloodstream infections, regulates its pathogenic capability in this way. We found that the ancestral transcriptional regulator YhaJ coordinately controls the genetically unlinked processes of fimbrial adhesion and tryptophan biosynthesis to maximize UPEC survival during infection of the bloodstream. These findings reveal insights into the underlying basis of UPEC pathogenesis and shed light onto how repurposing preexisting genes and regulators can enhance the efficacy of pathogens during systemic infection.

Bacterial genomes are highly dynamic and mosaic in nature. Even within a species, the content of a genome can vary dramatically. The genomic diversity in the *Escherichia coli* species has long been appreciated, with previous genome comparisons identifying <40% shared genes between selected prototype strains and more recent large-scale studies suggesting a staggeringly large pangenome of >50,000 genes (1, 2). Furthermore, the choice of which genes to preferentially express at any given time is unique to the lifestyle of a particular isolate, whereby the characteristics of the environment impact on how expression of the genome is regulated (3, 4). Bacterial cells typically function under the control of many regulatory layers to maximize fitness. The most basic of these is regulation at the transcriptional level, where the expression of an isolate’s genes must be coordinated in response to their environment. Transcriptional regulation is driven by the activity of transcription factors (TFs), regulatory proteins that are often responsive to specific environmental stimuli and modulate the activity of RNA polymerase at target promoters (5, 6). Bacteria typically encode many TFs to cope with the regulatory requirements of their lifestyle. For example, *E. coli* strains encode ~300 TFs (~6% of its genome dedicated to regulatory roles) highlighting the importance of this process (7). However, only a handful have been studied in detail, mostly within nonpathogenic *E. coli* K-12 strains, leaving the majority of these TFs and the roles that they play in clinically relevant pathogens largely unknown.

Uropathogenic *E. coli* (UPEC) is the most common cause of urinary tract infection (UTI), responsible for ~75% of all uncomplicated cases (8). One in four women suffering from UTI will experience a recurrent infection, and this is supported by the knowledge that UPEC isolates are adept at colonizing the gut asymptomatically, acting as a within-host reservoir for extraintestinal infection (9–13). Additionally, the increasing prevalence of antimicrobial resistance among UPEC isolates makes treatment of such infections problematic (14). Another major clinical concern is the ability of UPEC isolates to breach the kidney parenchyma during ascending UTI, which ultimately leads to bloodstream infection (BSI) (15). *E. coli* is the leading cause of BSI, which can develop into potentially lethal sepsis. Sepsis is a pressing global health issue, with ~50 million cases being reported worldwide in 2017 and a one in five mortality rate (16). Furthermore, BSI is the second most dominant syndrome related to antimicrobial resistance, being responsible for ~400,000 of the ~1.3 million deaths directly attributable to antimicrobial resistance in 2019 (14). UPEC are therefore capable of occupying multiple, distinct niches which ultimately requires the right combination of genes to be expressed to enhance fitness of the pathogen at such dissimilar host-sites (17, 18). Recent studies have successfully employed forward genetic screens to identify candidate genes important for UPEC BSI and UTI, revealing both shared and unique for fitness factors for each disease (19, 20). However, the underlying molecular mechanisms that drive BSI are largely unknown (15).

It is often assumed that conserved regulators within strains of the same species have the same regulatory functions. Indeed, TFs usually display an ancestral role in a narrow-spectrum process, such as the response to a specific environmental signal (21). Rewiring of transcriptional regulatory networks can lead to the emergence of new functions for a TF, and this process has been well explored between different bacterial species (22). However, our recent work has revealed that core encoded TFs with no known function can also play vastly different roles in gene regulation within distinct members of the same species (23, 24). This widespread regulatory flexibility leads to the control of gene expression that appears to be highly beneficial to the lifestyle of isolates that are often capable of occupying distinct ecological niches. For example, we revealed how the highly conserved *E. coli* LysR-type transcriptional regulator YhaJ controls specific and distinct gene sets within the gut pathogen enterohemorrhagic *E. coli* (EHEC) and the extraintestinal pathogen UPEC. In EHEC, YhaJ directly regulates expression of a pathogenicity island-encoded Type 3 Secretion System, essential for intestinal colonization and pathogenesis (23, 25). In contrast, YhaJ controls the expression of UPEC Type 1 fimbriae, a key adhesive structure that mediates binding to mannosylated glycans found on the bladder epithelium, via direct interaction within its phase-variable promoter region (26). Importantly, we also found that this regulatory flexibility was not restricted to horizontally acquired virulence genes and that core-genome encoded elements are also under strain-specific control. In many cases, this regulatory flexibility is driven by strain-specific binding dynamics of YhaJ, with only 15% of its total identified binding sites being shared between multiple strains. However, the TF-binding sequence is often identical between strains displaying regulatory flexibility, therefore the reasons for this variation are not always clear (27).

Here, we have identified that YhaJ controls a small regulon in UPEC consisting of genes individually required for full fitness during BSI. YhaJ activates expression of two virulence adhesion systems (Type 1 and F1C fimbriae) and tryptophan biosynthesis via both direct and indirect mechanisms. Exposure of UPEC to human serum but not urine enhanced expression of these systems, which were also up-regulated during colonization of the liver and spleen, indicating niche-specific regulatory control. Accordingly, deletion of *yhaJ* resulted in a fitness defect during murine BSI but had no detrimental effect on colonization of the kidney during ascending UTI. Importantly, individually deleting either the fimbrial or tryptophan biosynthetic gene clusters resulted in a comparable fitness defect during BSI. These results indicate that both adhesion to tissue and adaptive metabolism are individually required for systemic infection, but transcriptionally coordinated by a repurposed TF to maximize fitness within the host niche.

## Results

### The Highly Conserved TF YhaJ Is Required for UPEC Extraintestinal Infection.

**Fig. 1.**
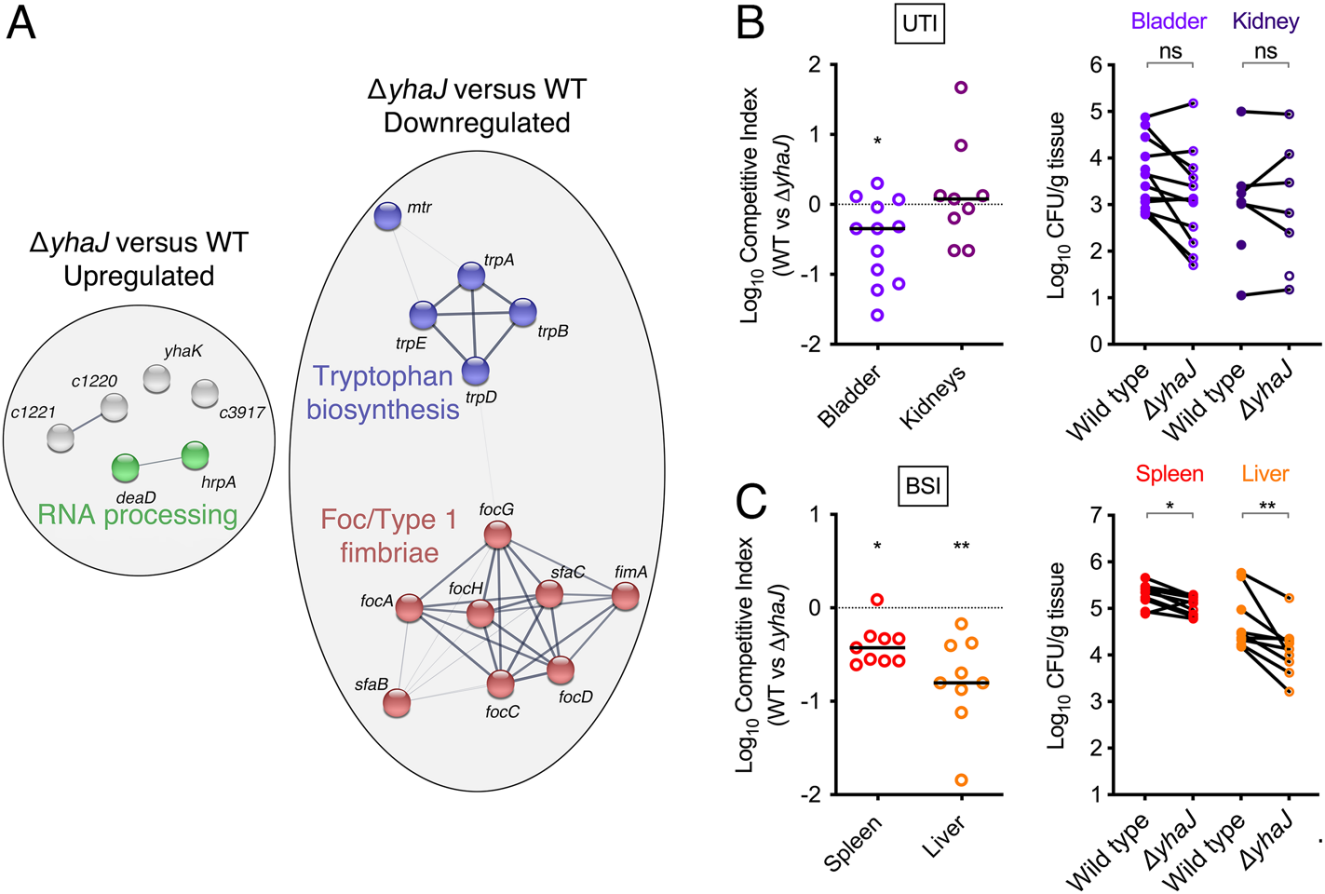
UPEC YhaJ controls a small regulon and contributes to extraintestinal pathogenesis. (*A*) STRING network identifying the functional groups of differentially expressed genes identified by RNA-seq analysis of WT UPEC versus Δ*yhaJ.* Connecting lines between the nodes indicate a known interaction between the corresponding encoded proteins. Nodes are color coded according to significantly identified functional groups. (*B*) Competitive index of WT UPEC versus Δ*yhaJ* during murine UTI. Mice were infected transurethrally with a 1:1 mixture of both strains. The data points indicate the fold decrease in Δ*yhaJ* CFU recovered per organ in comparison to WT UPEC CFU. The mean CFU per organ of each strain in each animal is indicated in the corresponding graph on the right. (*C*) Competitive index of WT UPEC versus Δ*yhaJ* during murine BSI. Mice were infected intravenously with a 1:1 mixture of both strains. The mean CFU per organ of each strain in each animal is indicated on the right. Statistical significance was determined using the Wilcoxon signed rank test for competitive index or the Wilcoxon matched-pairs signed-rank test for CFU comparison. *, ** and ns indicate *P* < 0.05, *P* < 0.01 or not significant respectively.

We previously discovered that the TF YhaJ, which is broadly conserved in >95% of *E. coli* strains, regulates a unique set of genes in UPEC that is distinct from intestinal isolates, including contributing to the direct control of Type 1 fimbriae via interaction with its phase-variable, invertible promoter element (23, 25). However, the role that YhaJ played during infection of distinct host niches remains elusive. To address this gap, we reanalyzed our previous transcriptomic data generated following culture of wild-type (WT) UPEC strain CFT073 versus a Δ*yhaJ* deletion mutant cultured in HEPES buffered Minimal Essential Media (MEM-HEPES) minimal medium to investigate the different ways in which this TF may impact on host fitness (23). In UPEC, YhaJ controls a small regulon consisting of 19 genes that are differentially expressed (False Discovery Rate (FDR) *P* < 0.05) in the mutant compared with the WT (Fig. 1*A*). STRING analysis broke these genes down into three discrete functional categories—upregulation of RNA processing (helicase encoding genes *hrpA* and *deaD*), downregulation of tryptophan biosynthesis (*trp* operon) and downregulation of F1C/Type 1 fimbrial adhesion (referred to as the *foc* and *fim* operons herein). Given that Type 1 fimbriae are a known UPEC virulence factor important for adhesion to bladder epithelial cells, we used a murine model of ascending UTI to test if YhaJ played a role in UPEC pathogenesis. The Δ*yhaJ* mutant displayed only a very modest and variable decrease in competitive fitness compared with the WT during a 1:1 coinfection of the bladder (*P* = 0.0161), with no significant difference in the mean bacterial burden per bladder (Fig. 1*B*). Furthermore, YhaJ was completely dispensable for competitive fitness during colonization of the kidney. This left the role of YhaJ and its regulated genes during host infection unclear. Interestingly, we noticed that genes related to *foc* fimbriae (*focD, focH*), *trp* biosynthesis (*trpA, trpB, trpD*), and RNA helicases (*hrpA, deaD*) had previously been identified as putative fitness factors in a transposon mutagenesis-based foreword genetic screen of UPEC BSI, but not UTI (20, 28). We therefore hypothesized that YhaJ may have a more important role during BSI, given its small regulon focused on these processes specifically. To test this, we assessed the competitive fitness of WT UPEC and Δ*yhaJ* during coinfection via tail-vein injection in a murine model of BSI*.* Strikingly, the Δ*yhaJ* mutant was significantly outcompeted by the WT and attenuated for colonization of the spleen (*P* = 0.0078; ~fivefold reduction in Colony Forming Units (CFU)) and liver (*P* = 0.0039; ~10-fold reduction in CFU) during BSI, displaying a consistent reduction in bacterial burden relative to the WT (Fig. 1*C*). These data suggest that YhaJ may play a more important role in infection of the bloodstream through the specific regulation of a discrete set of biological processes in UPEC.

### YhaJ Directly and Indirectly Controls Multiple BSI Fitness Factors.

**Fig. 2.**
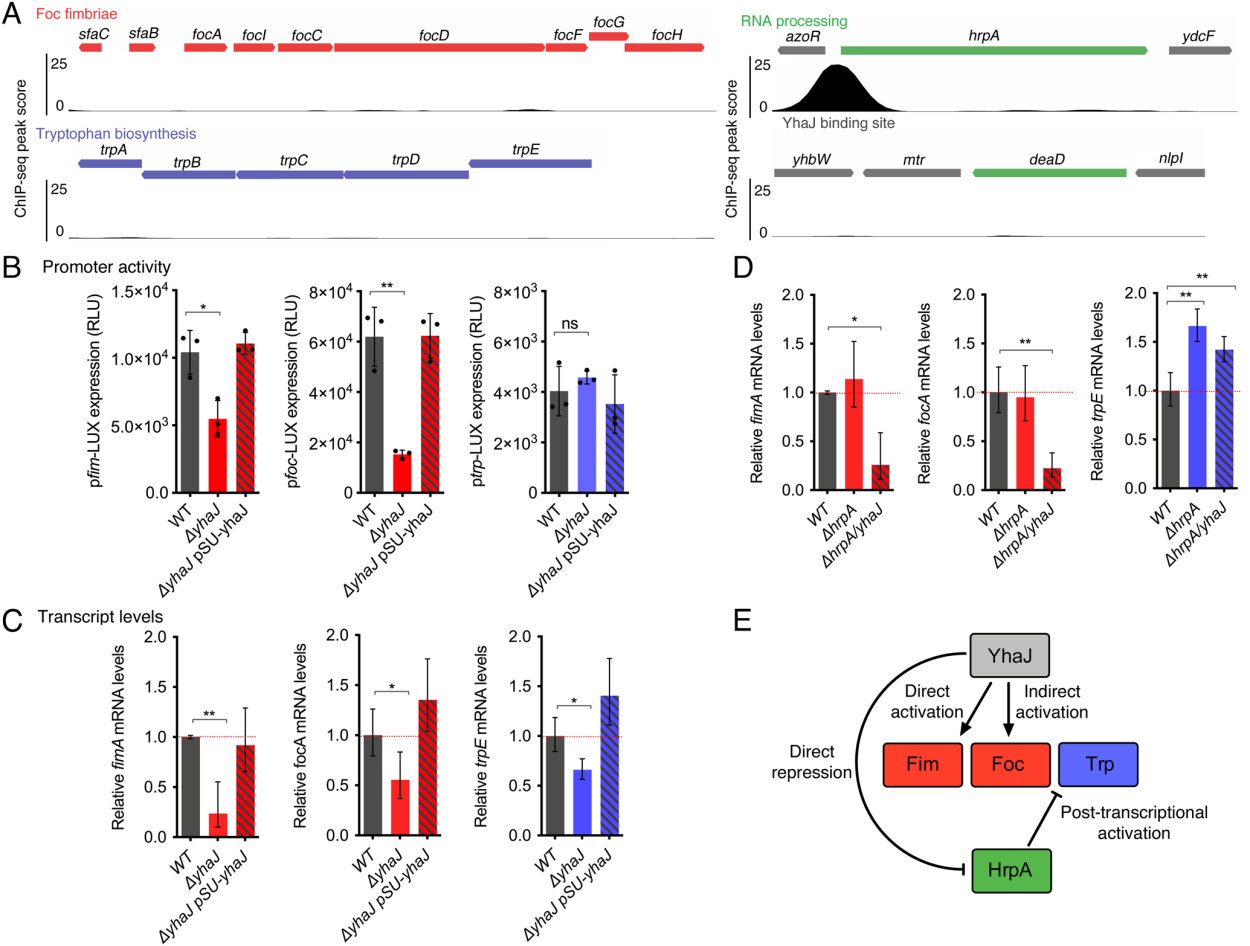
YhaJ controls multiple unlinked genes via direct and indirect mechanisms. (*A*) ChIP-seq plots of YhaJ global binding site analysis. Genes identified from the RNA-seq analysis are color coded according to functional group. (*B*) Transcriptional reporter assays to determine promoter activity in WT UPEC, Δ*yhaJ,* or Δ*yhaJ* pSU-*yhaJ* carrying either *fim, foc,* or *trp* promoter-LUX fusion plasmids. Data are depicted as relative luminescence units (RLU; luminescence flux/optical density). Reporter assays were performed in biological quadruplicate, and error bars represent SD. (*C*) qRT-PCR analysis of relative cellular transcript levels in the WT UPEC, Δ*yhaJ,* or Δ*yhaJ* pSU-*yhaJ* strain backgrounds. The red dotted line indicates the WT expression threshold. Data are derived from three biological replicates and error bars indicate SD. (*D*) qRT-PCR analysis of relative cellular transcript levels in the WT UPEC, Δ*hrpA,* or Δ*hrpA/yhaJ* strain backgrounds. For all analyses *, ** and ns indicate *P* < 0.05, *P* < 0.01 or not significant, respectively, as determined by the Student *t* test. (*E*) Model of the YhaJ regulatory network in UPEC. Arrows indicate activation whereas as blunt arrows indicate repression.

Our data suggested that YhaJ may be a key factor in UPEC’s ability to cause BSI through the regulation a small, discrete set of genes. Indeed, our previous work showed that YhaJ regulates *fim-*encoding genes in UPEC by binding directly to their phase-variable promoter region and positively influencing expression of Type 1 fimbriae (23). To investigate how YhaJ may enhance BSI fitness through the regulation of other factors such as *foc, trp,* and RNA helicases, we took a genetic approach. Inspection of the genome-wide YhaJ-binding pattern identified by ChIP-seq revealed a strong binding site directly upstream of the *hrpA* gene, encoding a DEAH-box RNA helicase (Fig. 2*A*) (27). Conversely, no YhaJ-binding site was identified upstream of the *foc* and *trp* operons, or DeaD helicase-encoding gene *deaD.* Electrophoretic Mobility Shift Assay (EMSA) analysis of these promoter regions using purified YhaJ verified these results (*SI Appendix*, Fig. S1). This finding suggests two scenarios—either YhaJ indirectly regulates these genes by influencing another TF or YhaJ plays a role in posttranscriptional regulation via HrpA. To test these hypotheses, we generated transcriptional reporter plasmids to assess activity of the *foc* and *trp* promoters and evaluated their relative transcript levels by qRT-PCR. As a control, we also assessed *fim* expression as we have previously observed the direct regulatory effect of YhaJ on its invertible promoter element. Deletion of *yhaJ* resulted in significant repression of both *fim* and *foc* promoter activity (~twofold reduction, *P* = 0.0157 and ~fourfold reduction, 0.0024, respectively), which could be completely complemented by expression of YhaJ in trans (Fig. 2*B*). This phenotype was mirrored in the qRT-PCR analysis of whole-cell mRNA levels (Fig. 2*C*). These data, taken with the lack of a YhaJ-binding site, suggest that *foc* is indirectly regulated by YhaJ at the level of transcription. Interestingly, we observed no change in activity of the *trp* promoter in the Δ*yhaJ* background but found that mRNA levels of the *trpE* gene (used to monitor *trp* expression) were significantly decreased and could be restored by complementation (*P* = 0.0228). This suggested that *trp* expression is indirectly regulated by YhaJ, via a posttranscriptional mechanism. RNA helicases, including HrpA, have previously been implicated in regulating transcript processing and degradation in multiple bacterial species (29, 30). We therefore hypothesized that the YhaJ-dependent regulation of *trp* may be mediated via HrpA. To test this, we evaluated *fim, foc,* and *trp* expression in Δ*hrpA* and Δ*hrpA*/*yhaJ* mutant backgrounds, noting that deletion of both *yhaJ* and *hrpA* did not affect the growth rate under the experimental conditions tested (*SI Appendix*, Fig. S2). As expected, neither *fim/foc* promoter activity nor transcript levels were affected in the Δ*hrpA* strain. In contrast, the regulatory defects observed in the Δ*hrpA*/*yhaJ* background were identical to those observed in the Δ*yhaJ* strain alone, supporting the hypothesis that *fim/foc* are controlled at the transcriptional level (Fig. 2*D* and *SI Appendix*, Fig. S3). Accordingly, the Δ*hrpA* mutant did not display a significant fitness defect relative to the WT during competitive murine BSI (*SI Appendix*, Fig. S4 *A* and *B*). In contrast, *trpE* mRNA levels were significantly increased in the Δ*hrpA* and Δ*hrpA/yhaJ* strains (*P* = 0.0036 and *P* = 0.0074 respectively). These results mirror what we observed in our RNA-seq analysis, in that deletion of *yhaJ* repressed *trp* but increased *hrpA* levels, suggesting that HrpA regulates *trp* transcript turnover. Thus, deletion of *hrpA* would incur the opposite effect (an accumulation of *trp* transcripts), possibly explaining the lack of an in vivo fitness defect as observed here. Taken together, these data have identified that YhaJ regulates its target genes through direct, indirect, and posttranscriptional mechanisms (Fig. 2*E*).

### Type 1 and F1C Fimbriae Are Controlled According to Host Ecological Context.

**Fig. 3.**
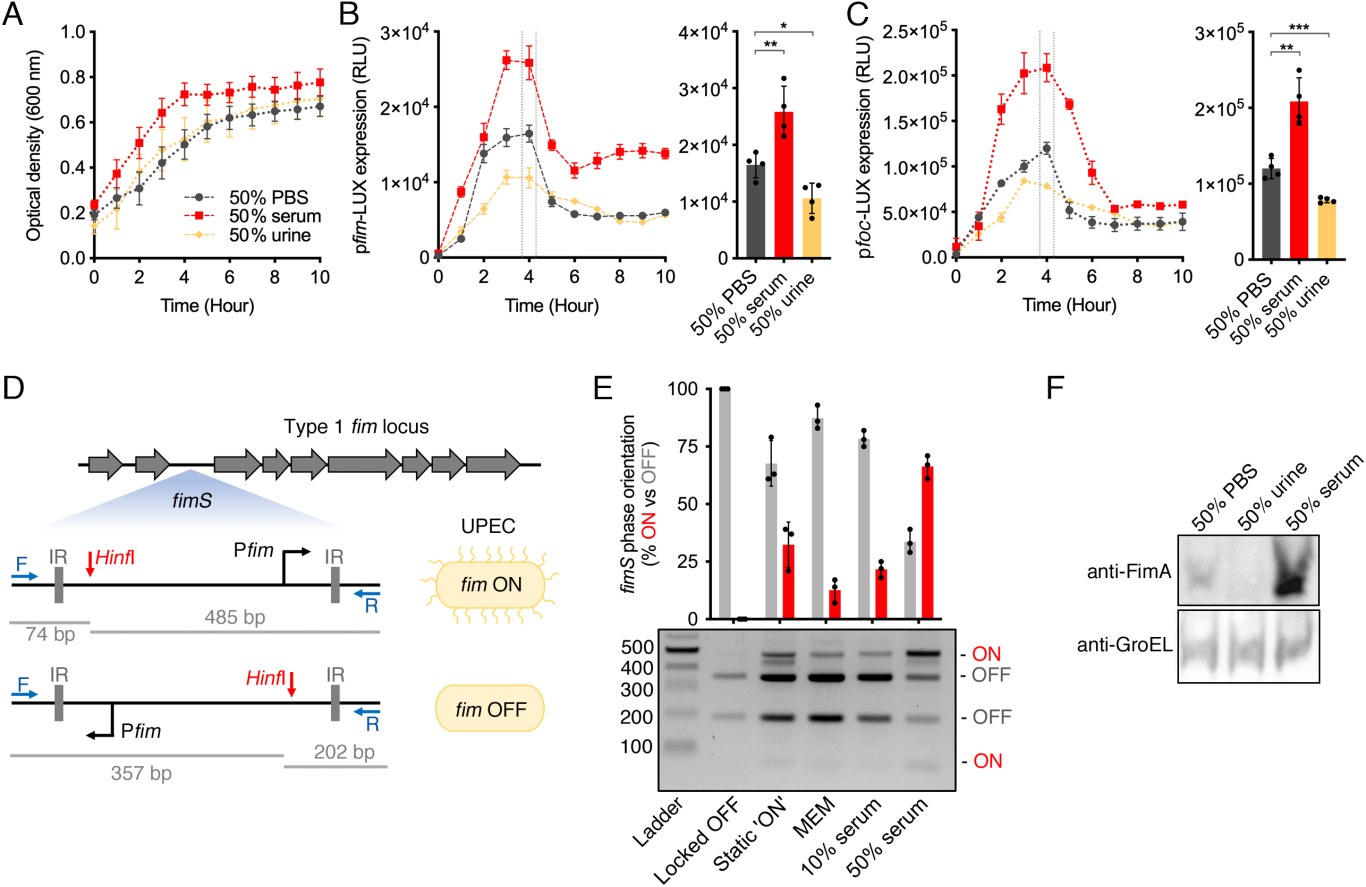
YhaJ-controlled virulence factors are inversely regulated by human serum or urine. (*A*) Growth of WT UPEC in MEM–HEPES supplemented with 50% human serum, urine or PBS as a negative control. (*B* and *C*) Transcriptional reporter assay of WT UPEC carrying a p*fim-*LUX or *pfoc-*LUX fusion plasmid grown in MEM–HEPES supplemented with 50% human serum, urine, or PBS. The graphs on the right illustrate data derived from the 4-h timepoint as indicated. Data are depicted as RLU. Reporter assays were performed in biological quadruplicate, and error bars represent SD. *, ** and *** indicate *P* < 0.05, *P* < 0.01 or *P* < 0.001 respectively as determined by the Student *t* test. (*D*) Schematic illustration of the phase variable Type 1 fimbriae promoter element *fimS.* This region is comprised of the *fim* promoter located between two inverted region (IR) regions. Amplification of *fimS by PCR* (priming sites in blue) results in a product containing a single *Hinf*I restriction site that can be digested to obtain DNA band sizes that represent the phase state of the element. (*E*) *fimS* phase orientation assay. DNA derived from UPEC population grown in static LB or MEM-HEPES (WT) supplemented with either 10 or 50% human serum digested with *Hinf*I and separated on a 1.5% agarose gel. A strain containing the *fimS* element locked in the OFF orientation was used as a control. The bars represent the relative proportion of *fimS* phase ON or OFF in each condition. The experiment was performed on three occasions with DNA obtained from independent cultures. (*F*) Immunoblot analysis of FimA expression levels from cultures of UPEC grown in MEM-HEPES supplemented with PBS, human urine or serum. GroEL levels were used as a loading control. Immunoblots were performed in biological triplicate.

Bacterial virulence factors, including fimbriae, are often regulated in response to signals perceived from the environment in order to modulate their expression appropriately (4, 31). Given the importance of YhaJ for fitness during murine BSI and its regulatory effect on distinct UPEC fimbrial genes, we hypothesized that these virulence factors may be uniquely regulated to benefit UPEC during BSI. To test this, we assayed growth and virulence gene expression (using the *fim* and *foc* reporters) in minimal media supplemented with either 50% human serum or urine to mimic the characteristics of the bloodstream or bladder environments. Medium supplementation with 50% Phosphate Buffered Saline (PBS) was used as a negative control. UPEC growth was enhanced in media supplemented with serum (specific growth rate of 0.46 ± 0.04 versus 0.26 ± 0.03) but not urine (specific growth rate of 0.28 ± 0.07) (Fig. 3*A*). Transcription of both *fim* and *foc* was significantly enhanced in response to serum (~1.5-fold increase, *P* = 0.0098 and ~twofold increase, *P* = 0.002 respectively), with the greatest fold changes in expression being observed at the mid-to-late exponential phase of growth (Fig. 3 *B* and *C*). Additionally, we verified the transcriptional reporter results using qRT-PCR (*SI Appendix*, Fig. S5). Intriguingly, urine supplementation in the media had the opposite effect to that of serum with a modest (~0.7-fold decrease), yet significant, repression of *fim/foc* being observed (*P* = 0.0153 and *P* = 0.0009, respectively). Note that Δ*yhaJ* retained the growth advantage induced by the presence of serum, as well as increased transcription of *fim* and *foc* in response to serum despite their expression levels being significantly lower in this genetic background (*SI Appendix*, Fig. S6 *A* and *B*). However, expression was not maximally restored to that of the WT in response to serum. This suggests that *fim* and *foc* are enhanced by YhaJ in concert with an independent stimulatory effect of serum on their expression levels and the more general growth-promoting effect of serum in minimal medium.

To validate the effects of serum on virulence gene expression, we next assessed the phase state of the *fim* promoter region in response to serum. The *fim* promoter is contained within an invertible element termed *fimS* (the *fim* switch) that upon stimulation by TFs can be orientated into an ON or OFF position (including YhaJ, which stimulates the ON orientation), signifying expression, or repression, of Type 1 fimbriae (32). The *fimS* element contains a unique *HinF*I restriction site, and this allows the overall phase state of a cell population to be determined using a simple PCR digestion assay (Fig. 3*D*). DNA corresponding to *fimS* was amplified from UPEC cultures grown in minimal media alone or supplemented with different concentrations of human serum. Digestion of this PCR product (559 bp) with *Hinf*I resulted in banding patterns that correspond to the orientation of the promoter, that is 74/485 bp for ON or 202/357 bp for OFF. Approximately 15% of the amplicons derived from UPEC cultured in minimal medium alone were phase ON under these conditions (Fig. 3*E*). As a negative control, we analyzed DNA from an *E. coli* strain (TUV93-0) that contains a mutation in *fimS* rendering the element permanently locked OFF. As a positive control for phase switching, we also cultured UPEC statically in LB media for 24 h, conditions known to induce the ON phase. This increased the ON proportion of the population to ~35%. Supplementation of minimal media with either 10 or 50% human serum increased the proportion of *fimS* phase on cells to ~20 and ~70%, respectively, in line with the gene expression data. Finally, we performed immunoblot analysis on lysates of UPEC cells cultured for 4 h in MEM–HEPES supplemented with PBS, urine or serum using antibodies specific for FimA, the major pilin subunit of Type 1 fimbriae. Growth in the presence of serum resulted in a marked increase in FimA production, whereas urine resulted in decreased protein expression (Fig. 3*F*). These results validate the transcriptional reporter assays and altogether suggest that human serum provides signals that enhance UPEC virulence gene expression.

### Type 1/F1C Fimbriae Are Up-Regulated during Murine BSI and Required for Systemic Organ Colonization.

**Fig. 4.**
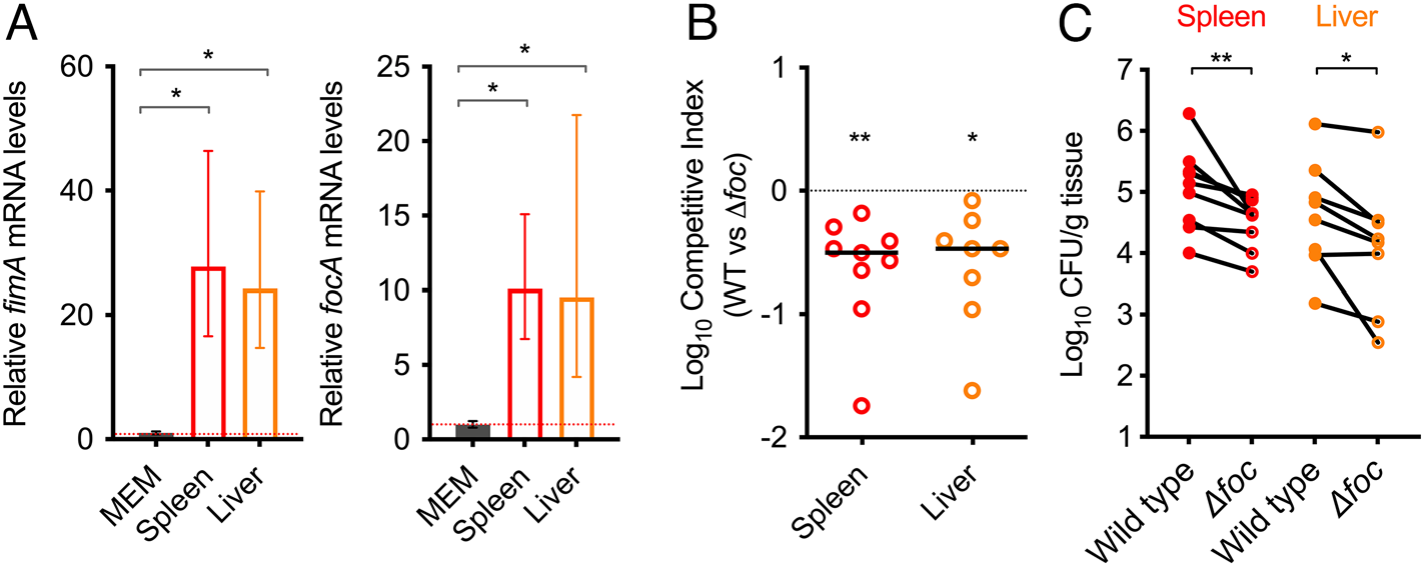
YhaJ-regulated virulence factors are up-regulated in vivo and required for full fitness during BSI. (*A*) qRT-PCR analysis of relative *fimA* and *focA* transcript levels in RNA derived from WT UPEC cultured in MEM–HEPES or infected murine tissue (spleens and livers) obtained from the BSI model. The red dotted line indicates the WT expression threshold. Data are derived from three biological replicates, and error bars indicate SD. * indicates *P* < 0.05 as determined by the Student *t* test. (*B*) Competitive index of WT UPEC versus Δ*foc* during murine BSI. Mice were infected intravenously with a 1:1 mixture of both strains. The data points indicate the fold decrease in Δ*foc* CFU recovered per organ in comparison to WT UPEC CFU. * or ** indicate *P* < 0.05 *P* < 0.01 as determined by the Wilcoxon signed rank test. (*C*) The mean CFU per organ of each strain in each animal. * or ** Indicate *P* < 0.05 *P* < 0.01 as determined by the Wilcoxon matched pairs signed rank test.

The data thus far revealed that human serum has a positive stimulatory effect on UPEC virulence gene expression, and that this may represent a functional switch required by the bacteria once the bloodstream has been encountered. During BSI, UPEC quickly associate with multiple, distinct organs, and perfusion of such tissue postmortem fails to lower the bacterial burden indicating colonization rather than transient association (33). This suggests that a role for fimbriae may be to adhere specifically to such host tissues during BSI. To investigate this*,* we isolated mRNA from livers and spleens of mice infected with WT UPEC in the murine BSI model. qRT-PCR analysis of both *fimA* and *focA* levels (representing the *fim* and *foc* operons, respectively) in vivo compared with growth in minimal media revealed that *fim* was significantly up-regulated by ~25-fold in both the spleen and liver (*P* = 0.035 and *P* = 0.048, respectively), whereas *foc* was induced ~11- and ~26-fold (*P* = 0.026 and *P* = 0.015) (Fig. 4*A*). This mirrors the results observed during growth in serum-supplemented media, suggesting that fimbriae are specifically induced in the host systemic environment and may promote colonization of systemic host organs. In a previous study, the Type 1 fimbriae were found to be required for full fitness of UPEC during murine BSI (33). We therefore hypothesized that there would be a similar dependency on *foc,* given its regulatory control by YhaJ and enhanced expression in vivo. To test this, we generated mutant deleted for the entire *foc* operon (Δ*foc*) and infected mice in a 1:1 ratio with WT UPEC. Strikingly, we observed that the Δ*foc* strain was significantly outcompeted by the WT for colonization of both the spleen (*P* = 0.0039) and liver (*P* = 0.0142), displaying an average ~fivefold relative reduction in CFUs recovered from each organ after ~18 h of infection (Fig. 4 *B* and *C*). These data indicate that *foc* fimbriae are a critical virulence factor expressed by UPEC that are required for tissue colonization during BSI.

### UPEC Up-Regulates *trp* Biosynthesis Independently of Tryptophan Levels to Enhance Niche-Fitness during BSI.

**Fig. 5.**
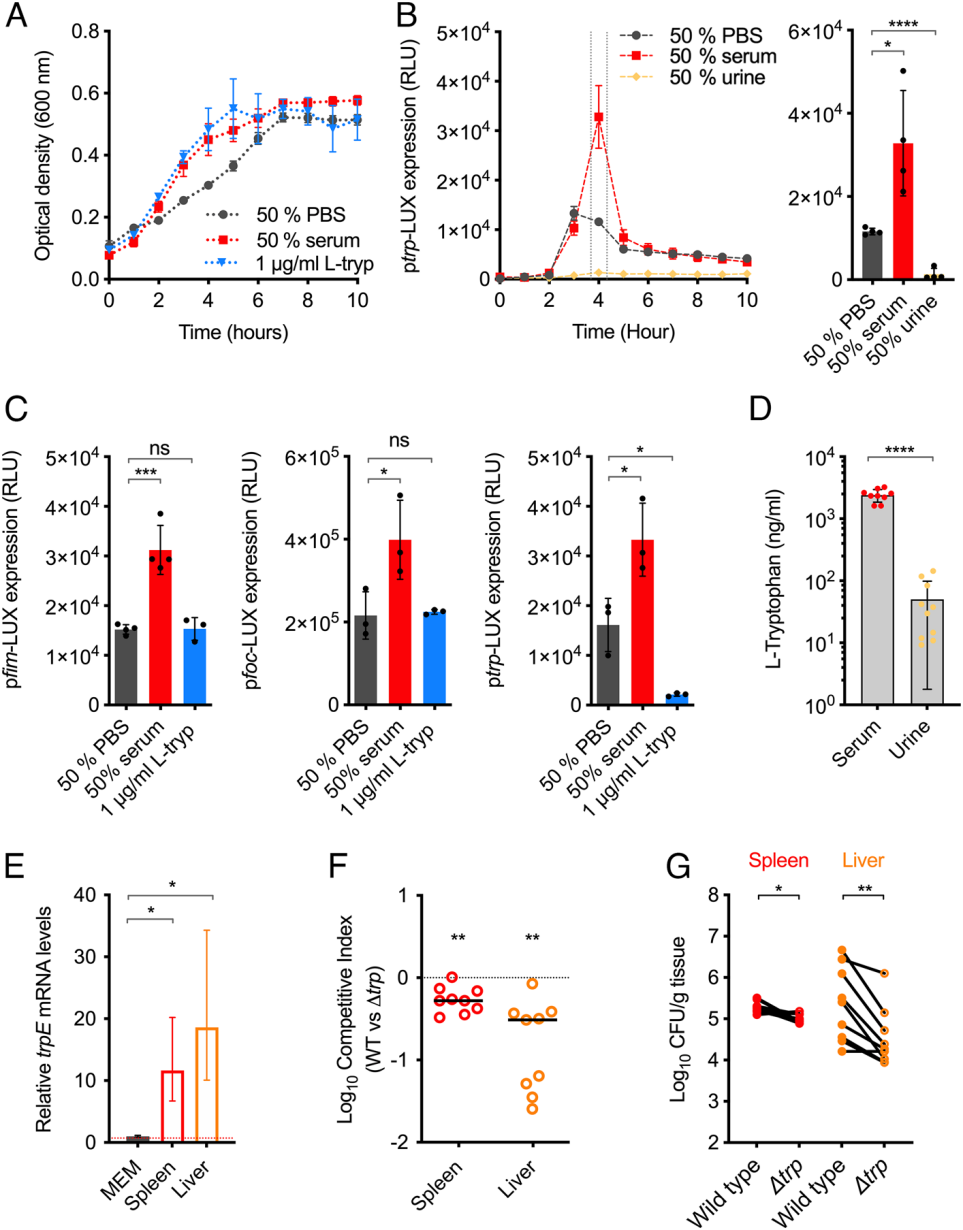
The BSI environment induces tryptophan biosynthesis to enhance UPEC fitness. (*A*) Growth of WT UPEC in MEM–HEPES supplemented with 50% human serum, 1 µg/mL L-tryptophan or 50% PBS as a negative control. (*B*) Transcriptional reporter assay of WT UPEC carrying a p*trp-*LUX fusion plasmid grown in MEM-HEPES supplemented with 50% human serum, urine or PBS. The graph on the right illustrated data derived from the 4-h timepoint as indicated. Data are depicted as RLU, assays were performed in biological quadruplicate, and error bars represent SD. * and **** indicate *P* < 0.05 or *P* < 0.0001, respectively, as determined by the Student *t* test. (*C*) Transcriptional reporter assays of WT UPEC carrying a p*fim-*LUX, *pfoc-*LUX, or p*trp-*LUX fusion plasmid grown in MEM–HEPES supplemented with 50% human serum, 1 µg/mL L-tryptophan or 50% PBS. *, *** and ns indicate *P* < 0.05, *P* < 0.001 or not significant respectively as determined by the Student *t* test. (*D*) Quantification of L-tryptophan levels from human serum or urine samples by UHPLC-QqQ-MS. **** indicates *P* < 0.0001 (Student *t* test). (*E*) qRT-PCR analysis of relative *trpE* transcript levels in RNA derived from WT UPEC cultured in MEM–HEPES or infected murine tissue (spleens and livers) obtained from the BSI model. The red dotted line indicates the WT expression threshold. Data are derived from three biological replicates, and error bars indicate SD. * Indicates *P* < 0.05 as determined by the Student *t* test. (*F*) Competitive index of WT UPEC versus Δ*trp* during murine BSI. Mice were infected intravenously with a 1:1 mixture of both strains. The data points indicate the fold decrease in Δ*trp* CFU recovered per organ in comparison to WT UPEC CFU. * or ** indicate *P* < 0.05 *P* < 0.01 as determined by the Wilcoxon signed rank test. (*G*) The mean CFU per organ of each strain in each animal. * or ** indicate *P* < 0.05 *P* < 0.01 as determined by the Wilcoxon matched pairs signed rank test.

Pathogen success in specific host niches is not solely driven by virulence factors, but also enhanced fitness via the regulation of metabolism. We therefore hypothesized that *trp* biosynthesis may be a key beneficial trait for UPEC during BSI that is regulated in response to host signals. Growth of UPEC in minimal media supplemented with 1 µg/mL L-tryptophan or human serum resulted in similar levels of growth enhancement (Fig. 5*A*). To understand if *trp* is regulated in response to host signals, we tested our reporter strain during growth in minimal media supplemented with 50% human serum. We observed an acute increase in *trp* expression (~threefold, *P* = 0.0156) at late exponential phase, comparable to that observed for *fim/foc* (Fig. 5*B* and *SI Appendix*, Fig. S5). In addition to serum, we also tested the response of the *fim, foc,* and *trp* reporter strains to L-tryptophan supplementation alone. There was no effect of L-tryptophan on the expression levels of *fim* or *foc,* and as would be expected *trp* levels were significantly repressed (>10-fold reduction, *P* = 0.0106) in the presence of excess L-tryptophan in the media (Fig. 5*C*). This was an important result, as it demonstrated that the growth advantage of L-tryptophan supplementation, and associated repression of *trp,* coincided with the opposite regulatory effect observed for serum supplementation (enhanced growth and *trp* expression). Therefore, it is plausible that a constituent of serum stimulates such changes in gene expression independently of L-tryptophan.

Interestingly, *trp* expression was strongly repressed in the presence of human urine (*P* < 0.0001), with levels of transcription undetectable for the entire course of the growth curve (Fig. 5*B*). This result suggested that urine may contain higher levels of tryptophan than those found in the bloodstream, thus alleviating the requirement of UPEC to synthesize this amino acid endogenously. To test this, we quantified the levels of L-tryptophan in serum and urine by ultra-high-performance liquid chromatography coupled with triple quadrupole mass spectrometry (Fig. 5*D*). The average concentration of L-tryptophan in serum was ~2,400 ± 556 ng/mL, which was ~48-fold higher than that of urine (50 ± 48 ng/mL). This suggested *trp* expression is triggered in serum, despite the high levels of L-tryptophan it contains. qRT-PCR analysis of samples cultured in minimal media spiked with human serum after 3 h of growth confirmed the transcriptional response observed above. Importantly, sampling the culture temporally after serum exposure revealed a significant increase in *trpE* transcription within 15 min (*SI Appendix*, Fig. S7). This rapid response to serum was also observed for *fimA* and *focA*, supporting the hypothesis that factors within the bloodstream provide potent regulatory triggers for UPEC genes. To more directly assess if *trp* biosynthetic genes were stimulated in response to the host during BSI, we measured *trp* expression levels in infected organs. qRT-PCR analysis revealed that the *trpE* gene (representing *trp* operon expression) was significantly up-regulated ~11 and ~18-fold in spleens and livers infected with UPEC compared with growth in minimal media (*P* = 0.047 and *P* = 0.038, respectively), supporting the hypothesis that *trp* expression is regulated by host-derived signals (Fig. 5*E*). To test if *trp* biosynthesis is required as a fitness factor for UPEC in vivo, we generated a deletion mutant of the entire *trp* operon (Δ*trp*) and coinfected mice with WT UPEC at a 1:1 ratio. The Δ*trp* strain was significantly outcompeted by the WT in both the liver and spleen of infected mice (*P* = 0.0039 and *P* = 0.0078, respectively), displaying an average ~fivefold decrease in CFUs recovered from either organ after 18 h of infection (Fig. 5 *F* and *G*). These data suggest that *trp* biosynthesis, triggered in response to the host, is required to maximize UPEC fitness during BSI, and that this trait is not dependent upon, but enhanced by the TF YhaJ.

## Discussion

Gene regulation is a central feature of how bacteria adapt to new environments, allowing exploitation of the local conditions (3, 24). This is particularly important for pathogens that can occupy and move between multiple niches within the host, whereby key genes important for each site must be actively regulated. Bacteria encode hundreds of TFs that collectively control global gene regulation (7). However, the individual intricacies of these networks are far from understood and widespread intraspecies variations in TF regulatory networks has emerged as an important theme in bacterial genetics (24). Here, we have identified that the highly conserved TF, YhaJ, controls a small gene set specifically in an extraintestinal pathogen that enhance fitness following infection of the host bloodstream. YhaJ activates expression of two adhesion systems (Type 1 and F1C fimbriae) and tryptophan biosynthesis by distinct mechanisms in UPEC, but not in an intestinal strain. Deletion of either of these systems, or *yhaJ* itself, results in a fitness defect during murine BSI. Furthermore, these independent systems are transcriptionally induced by host signals, and enhanced by the action of YhaJ, indicating layers of regulation that ultimately converge on UPEC ability to infect the bloodstream and associated systemic organs.

Niche adaptation underpins the ability of pathogens to colonize the host and cause infection (34). The UPEC pathotype is adept at competitively colonizing the gut where it does not cause disease but rather establishes a reservoir for potential systemwide dissemination (10, 11). However, what defines a UPEC isolate genetically has been a long-standing question in infection biology (1). Unlike gut pathotypes that often rely on discrete virulence factors to cause disease (such as the Type 3 Secretion System of EHEC), UPEC isolates typically encode a suite of genes that are not individually essential but collectively facilitate fitness during infection of the bladder (8). These include virulence factors such as Type 1 fimbriae that specifically mediate epithelial attachment to mannosylated glycan receptors found exclusively in the bladder (26, 35). Additionally, while Type 1 fimbriae have only recently been implicated in ascending upper UTI, some UPEC isolates encode P fimbriae that are important factors mediating colonization of the kidney (36–38). However, the ability of UPEC to cause a UTI extends beyond the expression of virulence factors. UPEC can also use alternative metabolism to take advantage of the limited nutrients available in urine (17). For example, ethanolamine has recently been identified as a key energy source during experimental UTI (39). Additionally, our group and others have previously shown that catabolism of D-serine, the most abundant amino acid in urine, confers a fitness advantage to UPEC enabling its exploitation as a source of energy, whereas intestinal pathotypes have lost this metabolic capacity thus restricting them to the gut niche (40–42).

While much research has been dedicated to understanding the mechanisms driving UTI, far less is known about the pathogenesis of BSI (8, 15). Clearly, invasion of the bloodstream followed by colonization of many distinct organs such as the liver and spleen present unique environmental and host-derived challenges, distinct to that of the bladder or kidney. It is well known that UPEC isolates capable of causing BSI are highly resistant to the bactericidal effects of serum (43). However, the metabolic and virulence requirements of UPEC during BSI are poorly understood. A small number of pathways, such as cell envelope homeostasis, capsule production, iron uptake and Type 1/P fimbriae, were previously identified as being important for murine BSI (33, 44). Additionally, many other potential fitness factors that are important for murine spleen and liver colonization have been identified by two groups employing transposon mutagenesis-based screening (28, 44). These studies revealed several potential, niche-specific, UPEC factors that had not been identified during a similar forward genetic screen using a murine UTI model (20). As is often the case with independent genetic screens, each BSI study identified overlapping fitness genes as well as candidates not revealed in the other. While the YhaJ-regulated genes identified here (*trpABD, focDH, hrpA*) were only identified as fitness factors by Subashchandrabose et al., several genes related to the same biological processes were underepresented in the screen by Hullahalklki and Waldor but did not reach statistical singificance in their analysis. Importantly, all the YhaJ-regulated biological processes identified by us using RNA-seq (F1C/Type 1 fimbriae, RNA processing, *trp* biosynthesis) were commonly identified as potential BSI fitness factors in the former study and validated individually here, revealing gene regulatory mechanisms that contribute to UPEC success during infection (23).

The regulation of Type 1 fimbriae is a well characterized process. Many TFs (including YhaJ) act on its phase-variable, invertible promoter to collectively control its expression state (31). Additionally, environmental signals such as temperature, pH, and nutrients are also known to stimulate regulation of *fim,* while *foc* regulation is poorly characterized. We found here that growth in minimal medium containing human urine decreases the expression of *fim* and *foc,* which is in line with a previous report on the repressive effects of urine on Type 1 fimbriae expression (45). In contrast, we observed an increase in both *fim* and *foc* expression in response to human serum supplementation to the media, indicating that introduction to the bloodstream environment may act as a trigger switch to promote fimbriae expression. Exposure of UPEC to serum has been previously reported to induce transcriptional changes, with one study observing a decrease in Type 1 fimbriae transcription (46, 47). However, it has also been reported that growth in serum and intraperitoneal infection of mice, induces expression of S-fimbriae (a highly homologous system to F1C), promoting survival in vivo (48). There are several possible explanations for these apparent contradictions, such as differences in growth conditions, methods of serum exposure, and potentially strain-specific responses. Regardless of these distinctions, we observed here that *fim*/*foc* are up-regulated by exposure to serum in vitro, as well as in vivo and play a role in systemic organ colonization by UPEC during murine BSI. Collectively Type 1 and F1C fimbriae are capable of binding to several host glycan-derived receptors expressed on bladder or kidney epithelial cells, including mannosylated uroplakins (Type 1), and galactosylceramides or globotriaosylceramides (F1C) (35, 36, 49, 50). Accordingly, Type 1 fimbriae are an important virulence factor required for bladder colonization, while F1C fimbriae play a role in attachment to the collecting ducts and distal tubules of the human kidney, as well as murine gut colonization by the probiotic strain *E. coli* Nissle 1917 (51, 52). Given the number of tissue types that may be encountered after systemic dissemination, it is possible that UPEC takes advantage of specific fimbriae–organ interactions to provide a colonization advantage during BSI (33). In addition, it has also been reported that fimbriated cells can promote intracellular survival during phagocytosis through specific macrophage adhesion events (53). This creates multiple scenarios in which UPEC could potentially benefit from positively regulating fimbrial adhesion during BSI. Furthermore, our work raises the possibility that other fimbriae types associated with UPEC disease, such as P fimbriae and Ucl fimbriae, which are located on pathogenicity-associated genomic islands, may also be subjected to complex regulatory control by conserved TFs located on the core chromosome (54–56).

Amino acid biosynthesis in bacteria is an important process, for the generation of critical cellular resources that may be in limited supply. Tryptophan is an essential amino acid in humans that can be efficiently synthesized by *E. coli* via the *trp* operon in response to low concentrations, facilitating growth on minimal medium containing only a single carbon and nitrogen source. We found that deletion of the *trp* locus resulted in a fitness defect during murine BSI. This agrees with a previous observation that *trp* biosynthesis is linked to fitness during BSI, but not during UTI (20, 28). Furthermore, it has also been identified that mutations in the shikimate biosynthetic pathway (the precursor steps to aromatic amino acid biosynthesis) confer a fitness defect to UPEC during BSI (28). In addition, we identified upregulation of *trp* in response to both human serum supplementation to growth media and the in vivo environment of the liver and spleen. In contrast, exposure of UPEC to human urine in the media strongly repressed *trp* expression. These results suggested that differences in amino acid availability may be a critical determinant of what constitutes a UPEC fitness factor, depending on the host niche that is encountered. In contrast with this hypothesis, we found that levels of tryptophan were actually higher in serum than urine, suggesting that while *trp* biosynthesis is indeed critical for pathogen success, it is regulated independently of exogenous L-tryptophan availability in the bloodstream. Amino acid biosynthesis has been identified as a key advantage to enteropathogens within the gut, as it allows them to overcome colonization resistance incurred by amino acid scavenging members of the microbiota (57). While the ecological context is distinct, convention could argue this molecular scenario mirrors the notion of nutrient competition with the host during BSI. Indeed, tryptophan is an essential amino acid in high demand by human cells, being required for synthesis of proteins and many critical bioactive molecules such as kynurenine, serotonin, melanin and tryptamine (58). Additionally, it has been reported that severe bacteraemic patients express higher activity of the enzyme indoleamine 2,3-dioxygenase, which converts tryptophan to kynurenine (59, 60). However, the bloodstream is rich in this amino acid, and our data showing enhanced growth in minimal media supplemented with L-tryptophan suggest that UPEC is capable of meeting its cellular demands at lower concentrations than those encountered in vivo. Thus, high levels of tryptophan found in serum should in principle repress *trp* biosynthesis. Nonetheless, our gene expression studies have identified that *trp* is indeed induced rapidly in response to serum and up-regulated during systemic organ colonization. This suggests that an unknown host-signal exists to trigger *trp* expression, and that there may be an alternative fate for synthesized L-tryptophan independent of nutritional demand. Alternatively, the functionality of *E. coli* L-tryptophan permeases (Mtr, AroP and TnaB) is unknown in the context of systemic infection, and therefore endogenous L-tryptophan biosynthesis may be required irrespective of its high concentration in serum. Furthermore, other factors such as complexation of L-tryptophan with serum albumin, estimated to account for ~85% of all circulating L-tryptophan, could impact on its free availability and negatively affect the interplay between amino acid uptake and synthesis by UPEC (58).

Coregulation of critical fitness genes provides a strategy by which UPEC can coordinate genetically distinct biological processes that collectively benefit its pathogenic lifestyle, without relying on the horizontal acquisition of new virulence factors. While the historical function of YhaJ is unknown, the rewiring of its role in the *E. coli* transcriptional regulatory network has revealed its critical function in coordinating gene expression (23, 27). Importantly, this process appears specific to the pathotype in question. In EHEC, YhaJ directly controls the expression of a pathogenicity island encoded Type 3 secretion system and several phage-encoded effectors. It also regulates expression of a D-serine transporter, YhaO, but this occurs independently of D-serine sensing (25). This reveals traits similar to those reported here for UPEC; coregulation of *fim, foc* and *trp* by YhaJ to provide benefit for survival during BSI, which is independent of an environmental trigger. While it is possible that YhaJ may respond to a yet unknown signal, it appears to be reprogrammed as a “regulatory aide” enhancing the expression of key genetic factors in distinct pathogens. This represents a clever strategy for bacteria in recycling preexisting, nonessential TFs to perform new beneficial roles (24). Additionally, the finding that YhaJ mediates indirect regulatory effects (i.e., activation of *foc via* an unknown factor and direct control of HrpA) creates layers of regulation that are not solely dependent on common environmental signals. Indeed, YhaJ directly controls expression of HrpA, a known RNA helicase with a role in transcript processing. Upon discovery of HrpA, it was found to promote transcript cleavage of F1845 fimbriae via an unidentified endoribonuclease (30). While we did not identify any role for HrpA in regulation of *fim* or *foc* levels, we provide evidence that *trp* may be in part controlled posttranscriptionally. Recently, novel roles for HrpA have been described in regulating global transcript levels in the Lyme disease pathogen *Borrelia burgdorferi* and contributing to its virulence in mice as well as a role in tolerance to antibiotics in *E. coli* (29, 61). The precise mechanisms of indirect regulation by YhaJ remain unknown, but our hypothesis is that YhaJ likely functions in concert with further transcriptional and posttranscriptional regulators to tailor fitness gene expression in UPEC.

## Conclusion

We have identified the highly conserved TF YhaJ as an important regulator of UPEC pathogenesis, through the coordinated control of several distinct fitness factors. This work expands the concept of intraspecies regulatory rewiring and reveals mechanisms of how an extraintestinal pathogen functions during infection of the bloodstream (24). These findings are important for understanding the fundamental processes of UPEC pathogenesis but also reveal targets that could potentially be exploited in the pursuit of alternative therapeutics to combat BSI. For example, fimbrial antagonists and inhibitors (termed pilicides) have shown promise in their ability to block pathogen adhesion to host cells (56, 62). Additionally, an allosteric small molecule inhibitor of *Mycobacterium tuberculosis* was recently discovered to function by blocking its tryptophan biosynthetic machinery and was successful in reducing virulence in a mouse model (63). Therefore, the ongoing exploration of pathogen regulatory networks can reveal critically regulated factors for targeting by alternative antimicrobials.

## Materials and Methods

A complete list of all bacterial strains, plasmids and primers used in this study is detailed in the *SI Appendix* (*SI Appendix*, Tables S1–S3). Detailed *Materials and Methods* of the experiments and analysis performed here are included in *SI Appendix*. This includes growth conditions, strain generation, cloning procedures, RNA-seq/ChIP-seq data analysis, qRT-PCR, mouse models, UHPLC-QqQ-MS, EMSA, phase variation assays, immunoblots, and data analysis. All raw sequencing data were obtained from the European Nucleotide Archive under the project accession number PRJEB12065.

## Supplementary Material

Appendix 01 (PDF)

## Data Availability

All study data are included in the article and/or *SI Appendix*. Previously published data were used for this work [RNA-seq/ChIP-seq data presented in Figs. 1*A* and 2*A* was previously generated by us and stored in the European Nucleotide Archive under the accession PRJEB12065. It was reanalysed and interpreted for the current study and no figures have been duplicated. The original citation has been clearly acknowledged in the manuscript: Connolly et al. (23).].
