# Supplementary material for: Distinct ecological fitness factors coordinated by a conserved Escherichia coli regulator during systemic bloodstream infection
Source: Proc Natl Acad Sci U S A. 2022 Dec 27;120(1):e2212175120. doi: 10.1073/pnas.2212175120 (PMC9910484; doi:10.1073/pnas.2212175120)
Supplement: Supplementary file 1 — Appendix 01 (PDF) [file pnas.2212175120.sapp.pdf]

Supplementary Information Appendix

Distinct ecological fitness factors co-ordinated by a conserved *Escherichia coli* regulator during systemic bloodstream infection

Nicky O'Boyle, Gillian R Douce, Gillian Farrell, Nicholas J W Rattray, Mark A Schembri, Andrew J Roe and James P R Connolly

This PDF includes:

Material and methods

Figures S1-S7

Tables S1-S4

Supplementary references

Materials and methods

Bacterial growth conditions

The strain CFT073 was referred to as UPEC throughout this study. All strains, derivatives and plasmids used are detailed in Tables S1 and S2. Overnight bacterial cultures of a single colony grown in 5 ml of LB (~16 hours) at 37°C with 200 RPM shaking were washed in sterile PBS and diluted 1/100 into LB, M9 minimal media or MEM-HEPES for bacterial growth assays. Pooled human serum was purchased from Life Science Group Ltd. Pooled human urine was obtained from healthy volunteers. Serum and urine were filtered, pre-warmed to 37°C and typically added at a concentration of 10 or 50 % to the indicated media. Control cultures contained an equal proportion of sterile PBS to serum or urine. Where indicated, serum was inactivated by heat treatment at 56°C for 30 minutes. All other chemicals and antibiotics were purchased from Sigma Aldrich.

Lambda Red mediated mutagenesis

Isogenic strains of *E. coli* containing single or multiple gene deletions were generated by Lambda Red recombineering (1). Briefly, resistance cassettes from pKD3 (chloramphenicol) or pKD4 (kanamycin) were amplified by PCR using primers (Table S3) flanked with 50 bp overhangs complementary to the sequence directly adjacent to the 5' and 3' ends of the gene of interest. PCR products were purified and concentrated by phenol:chloroform extraction followed by ethanol precipitation. The parental strain was transformed with pKD46 and grown overnight at 30°C in LB containing 100 μg/ml ampicillin. This was used to inoculate SOB containing 100 μg/ml ampicillin followed by culture at 30°C for approximately 2 hours (OD_{600} of ~0.2) before the addition of 10 mM arabinose to the cultures. After a further hour of growth, cells were chilled on ice for 5 minutes and harvested by centrifugation at 3,500 RPM for 5 minutes. The pellet washed five times in 1 ml of ice-cold distilled water before a final resuspension in ice cold water at 100X concentration relative to the starting culture volume. 50 μl of cells was electroporated with ~1 μg of PCR product and cells were recovered in 1 ml of pre-warmed SOC for 2 hours at 37°C. Half the reaction was plated out onto the appropriate antibiotic containing LB agar plates and incubated overnight at 37°C. The remaining mixture was left overnight at room temperature and plated the following day. Colonies

were screened by PCR using check primers flanking the deleted region to identify successful recombinants. Positive mutants were subsequently transformed with pCP20 (ampicillin, 30°) and re-streaked non-selectively at 42°C to remove the resistance cassettes. Clean deletions were confirmed by colony PCR.

Cloning procedures

Complementation and reporter plasmids were generated by standard cloning using restriction digestion/ligation. All primers are listed in Table S3. For complementation plasmids, genes were amplified by PCR from purified wild type genomic DNA using primers containing *BamH*I (5') and *Xba*I (3') overhangs. PCRs were then gel extracted, digested, phosphatase treated and ligated into pSU-PROM according to the manufacturer's specifications (2). This created constructs where the gene of interest was expressed constitutively from the *E. coli* Tat promoter. For reporter plasmids, promoter regions containing approximately 200 bp upstream of the gene of interest were amplified by PCR from genomic DNA using primers containing *EcoR*I (5') and *BamH*I (3') overhangs. PCRs were cloned into pMK1-LUX, as described above (3). This created transcriptional reporters for genes of interest where their respective promoters were fused to the *luxCDABE* cassette. For all cloning reactions, restriction enzymes and Q5 high fidelity polymerase were purchased from New England Biolabs, and T4 ligase was purchased from Invitrogen. All plasmid inserts were sequenced (Eurofins) to confirm accurate construct generation.

LUX-promoter fusion transcriptional reporter assays

Promoter activity was determined by measuring in parallel the cell density (OD_{600}) and absolute luminescence of cultures carrying LUX reporter fusions at the same phase of growth using a FLUOstar Omega plate reader (BMG Labtech). Assays were performed in white walled/clear bottom microtiter plates and relative luminescence units (RLU) were determined by dividing the absolute luminescence values by OD_{600}. Experiments were performed in biological triplicate and statistical significance determined using the students *t*-test.

RNA-seq and ChIP-seq analysis

RNA-seq and ChIP-seq data from our previous study was retrieved from the European Nucleotide Archive (project accession number PRJEB12065) and reanalyzed for this

study (4, 5). Raw fastq files were imported into CLC Genomics Workbench version 7 (Qiagen) and the reads were aligned to the CFT073 reference genome (NCBI accession number: NC_004431). For RNA-seq, differential expression was performed using the EdgeR tool implemented in CLC (6). Relative expression levels were determined as absolute fold change and genes were considered as significantly differentially expressed by a false-discovery rate corrected p-value threshold of ≤ 0.05. For ChIP-Seq, peaks were called using the shape analysis tool in CLC (p-value ≤ 0.05) and visually inspected as read tracks aligned to the reference, whereby a TF binding site is defined by a bimodal intersecting peak signifying forward and reverse strand reads aligning either side of the binding site (7). Network analysis of biological functional groups associated with differentially expressed genes and TF binding sites was carried out using the STRING tool (8).

Murine models of urinary tract and bloodstream infection

The UTI model was carried out essentially as described previously (9). The bacterial inoculum was prepared by resuspending twice sub-cultured overnight cultures of UPEC or mutant derivatives grown statically in LB media in sterile phosphate-buffered saline (PBS) to a final concentration of ~2 x 10^8 CFU/ml. Female 8-week-old C57BL/6 mice (Charles River) were inoculated trans-urethrally with 50 μl of the inoculum (~1 x 10^7 CFU) using a lubricated catheter. For co-infections of wild and mutant strains, inoculums for each were prepared identically and mixed 1:1 prior to administration to the animals. After 48 hours of infection, mice were euthanised humanely using increasing CO_2 and cervical dislocation.

The BSI model was carried out essentially as previously described (10). The bacterial inoculum was prepared by resuspending overnight cultures of UPEC or mutant derivatives cultured in LB media in sterile PBS to a final concentration of ~1 x 10^8 CFU/ml. Female 8-week-old BALB/c mice (Charles River) were restrained using a Braintree restrainer (Fisher Scientific). The inoculum was administered by tail vein injection using 100 μl of the inoculum (~10^7 CFU). For co-infections of wild and mutant strains, inoculums for each were prepared identically and mixed 1:1 prior to administration to the animals. After 18 hours of infection, mice were euthanised humanely using increasing CO_2 and cervical dislocation.

For CFU counts, relevant organs were removed using sterile forceps and scissors followed by placement in 2 ml sterile PBS. Tissues were homogenised using a handheld mechanical homogeniser (Fisher Scientific), serially diluted in sterile PBS and plated on LB agar at different concentrations to determine the total CFU/g of tissue. Competitive infection burden was performed by differentially plating the homogenates in parallel on non-selective and selective (antibiotic containing relative to the resistance cassette used for gene deletion) LB agar plates. The competitive index of each experiment was calculated by dividing the ratio of mutant to wild type UPEC in the homogenates by the ratio of mutant to wild type in the inoculum. Statistical significance for competitive infections was determined using the Wilcoxon signed-rank test (hypothetical value of 0) on log-transformed values determined by the above calculation or by Wilcoxon matched-pairs signed-rank test on CFU counts for each strain.

Ethics statement

All animal experiments were performed in strict accordance with the United Kingdom Home Office Animals Scientific Procedures Act of 1986 under the personal project licence numbers 70-8713 and PP1440270. The experiments were subject to local ethical approval and consideration given to the refine, reduce and replace principals wherever possible so as all efforts were made to minimize animal suffering.

Quantification of L-tryptophan by ultra-high-performance liquid chromatography coupled with triple quadrupole mass spectrometry (UHPLC-QqQ-MS)

Sample analysis was conducted using a Shimadzu Nexera ultra-high-performance liquid chromatography (UPLC) system (Columbia, MD, USA) equipped with two pumps (LC-40 D-XS), a column oven (CTO-40C) and an auto-sampler (SIL-40C X3). Peak resolution and separation for all samples were achieved by using a Discovery HS F5 3 PFPP column (2.1 mm I.D. x 150 mm L., 3 um - Sigma Aldrich P/N 567503). Mass spectrometric detection was performed on a Shimadzu LC-MS/MS 8060-NX triple quadrupole mass spectrometer system (Columbia, MD, USA), equipped with a dual ionization source operated in positive electrospray ionization mode and multiple reaction monitoring mode to achieve unit resolution. The multiple reaction monitoring (MRM) transitions and compound dependent parameters, such as voltage potential Q1, Q3, and collision energy (CE), are shown in Table S4. Optimized parameters were

obtained by the product ion scan mode of individual analytes (1 µg/mL – conc of standards used to develop method) at 0.4 mL/min in water/formic acid (99.9/0.01, v/v). Parameters for multiple reaction monitoring (MRM) detection in the POS mode were as follows: Nebulizer gas: 3.0 L/min; heating gas: 10 L/min; drying gas: 10 L/min; interface temperature: 300°C; desolvation line temperature: 250°C; heat block temperature: 400°C. Mass transitions were monitored at 5 ms dwell times and unit mass resolutions and individual parameters are listed in Table S4. The mobile phase consisted of a 0.1% Formic acid Water and 0.1% Formic acid Acetonitrile. The chromatographic separation was achieved using a 25 min gradient elution. Lab Solutions LCMS Ver. 5.109 (Shimadzu Corporation) was used for data collection and quantitation.

RNA extraction from bacterial cultures and infected tissue

For *in vitro* samples, 2 ml of bacterial culture was normalised to the same OD_{600}, centrifuged, and resuspended in RNAlater (Ambion). Total RNA was extracted from the cell pellets using a Monarch RNA extraction kit (New England Biolabs) according to the manufacturer's specifications. Residual genomic DNA was removed using TURBO DNase (ThermoFisher Scientific) followed by PCR validation. The RNA was concentrated using phenol:chloroform extraction followed by ethanol precipitation. For *in vivo* samples, 5 mm sections of infected liver and spleen were stored in RNAlater immediately after dissection for 24 hours at 4°C. The tissues were subsequently homogenised using a TissueLyser LT (Qiagen) and total RNA extracted using a mirVANA kit (Ambion) according to the manufacturer's specifications. Genomic DNA was removed using TURBO DNase. RNA samples were analyzed on a Qubit (ThermoFisher Scientific) and assessed for degradation using agarose gel electrophoresis.

Quantitative real time PCR (RT-qPCR)

RNA samples were normalised and cDNA synthesis performed using the LunaScript RT SuperMix kit (New England Biolabs) according to the manufacturer specification. RT-qPCR was performed on the resulting cDNA using a LightCycler 96 Real-Time PCR system (Roche) and the Luna Universal qPCR Master Mix kit (New England Biolabs). The reactions were performed in technical triplicate and each gene that was analysed was performed in biological triplicate. All genes were normalised against the

housekeeping gene, *gapA*. All primers used in RT-qPCR were checked for efficiency (90-110%) using standards made from template gDNA (100, 20, 4, 0.8 and 0.16 ng/μl). The data was then analysed using the $2^{-\Delta\Delta CT}$ method (11).

Type I fimbriae phase variation assay

A PCR based approach to determine the proportion of a cell population expressing Type I fimbriae (*fim*) was carried out as previously described (4). Briefly, 2 μl of bacterial culture was added to 8 μl of nuclease free water and boiled. This solution was used as a PCR template for amplification of the *fim* phase-variable promoter region. The PCR product was purified using an NEB Monarch kit and digested with *Hinf*I before being separated on a 2 % agarose gel by electrophoresis. The promoter region contains a unique *Hinf*I restriction site and digestion of the PCR product (559 bp) results in a banding pattern of 74 bp and 485 bp (*fim* phase ON) or 202 bp and 357 bp (*fim* phase OFF). Relative band density was determined using ImageJ. The *fim* promoter region from *E. coli* TUV93-0 was used as a negative control as it is permanently in a locked OFF state.

Immunoblot analysis

Samples (1 ml) of bacterial were normalised for cell density and harvested by centrifugation. Pellets were resuspended in 100 μl 4x LDS sample buffer (Thermo Fisher), acidified with concentrated HCl to de-polymerise FimA subunits and boiled for 10 minutes before being neutralised with 10 M NaOH. 20 μl of lysate was separated by SDS-PAGE on a 4-12 % Bis-Tris NuPAGE gel (Thermo Fisher) at 180 volts for 45 minutes before being transferred to a 0.45 μm nitrocellulose membrane (GE Healthcare) at 30 volts for 90 minutes. Membranes were blocked with 5 % skim milk powder in PBST followed by incubation with primary (anti-FimA and anti-DnaK) and secondary antibodies (anti-Rabbit HRP-conjugated), separated by 3x 10 minute PBST washes. Immunoblots were incubated with SuperSignal West Pico chemiluminescent substrate for 5 minutes before imaging using the G:Box Chemi system (Syngene).

Recombinant YhaJ purification

6x Histidine tagged YhaJ cloned into pET28 was overexpressed in *E.coli* BL21-DE3 cells using 1 mM IPTG and purified as previously described (12). Cell pellets were

resuspended in wash buffer (200 mM NaCl, 50 mM Tris, 40 mM Imidazole, 10% glycerol) and lysed by French press. YhaJ was immobilised on a HisTrap column (GE Healthcare) using an AKTA-prime and purified by size-exclusion using a Superdex S200 column (GE Healthcare).

Electrophoretic mobility shift assay (EMSA)

EMSA analysis was performed as previously described (4). Promoter regions of interest were amplified by PCR and ddUTP-11-DIG labelled using the DIG Gel Shift Kit (Roche). Binding reactions were set up in 20 μl using 0.2 ng/μl of labelled DNA with increasing concentrations of purified YhaJ (0, 0.3, 0.6 and 1 μM) for 45 minutes at room temperature. Reactions were separated on a 6 % DNA retardation gel (Invitrogen) and transferred to a positively charged nylon membrane (Roche) using the NOVEX system (Thermo Fisher). Transferred membranes were UV crosslinked, blocked, and then probed with AP conjugated anti-DIG antibody at 1/10000. EMSAs were imaged on a ChemiDoc imaging system (Bio-Rad). EMSAs were performed in triplicate.

Statistical analysis

ChIP-seq and RNA-seq analysis was performed using CLC Genomic Workbench version 7.5 (Qiagen). All other statistical analyses (students *t*-tests, Mann-Whitney *U*-tests and Wilcoxon signed rank tests) were performed using GraphPad Prism version 8.

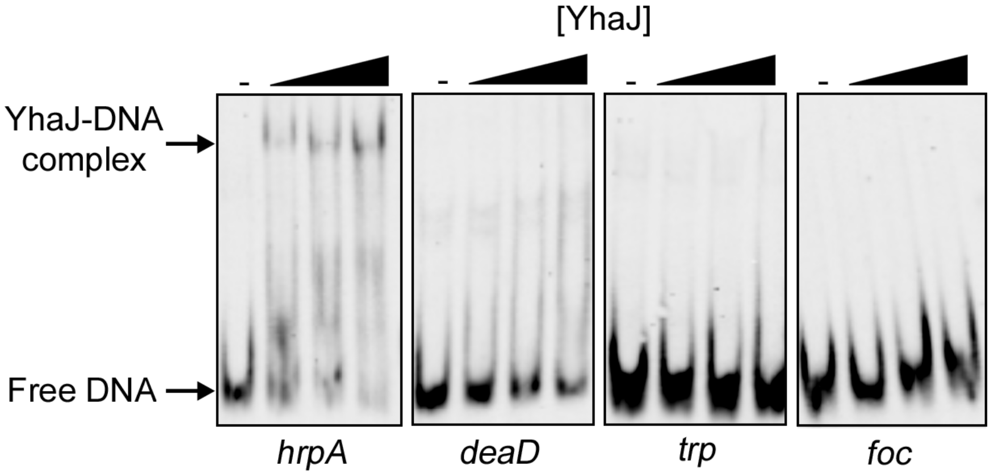

Fig S1. Validation of YhaJ binding to target promoters. EMSA analysis of purified recombinant YhaJ interacting with purified DNA corresponding to the *hrpA, deaD, trp* and *foc* 5’ regulatory regions. The gradient indicates increasing concentrations of YhaJ relative to a fixed concentration of DIG-labelled DNA probe.

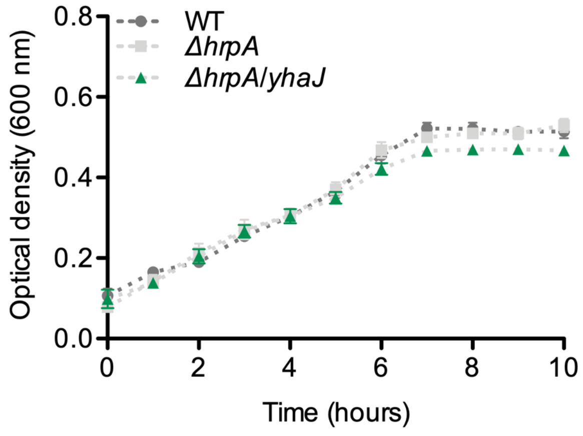

Fig S2. Growth of WT UPEC, Δ*hrpA* and Δ*hrpA/yhaJ* in MEM-HEPES minimal media.

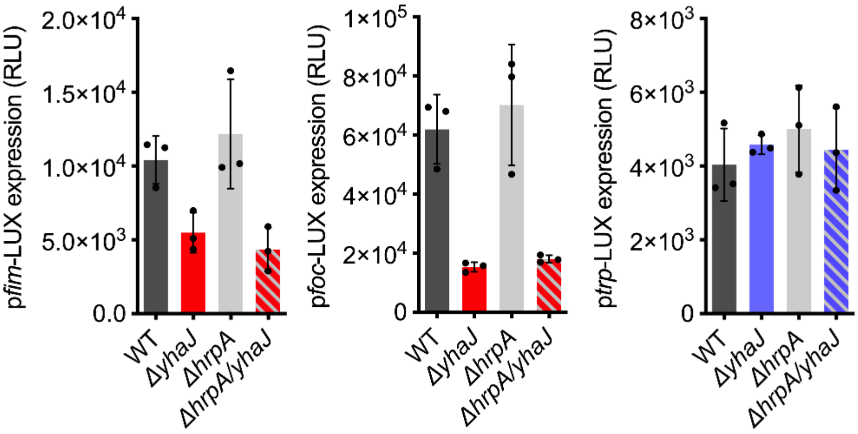

Fig S3. Transcriptional reporter assay of WT UPEC, Δ*yhaJ,* Δ*hrpA* and Δ*hrpA/yhaJ* strains carrying a p*fim*-LUX, p*foc*-LUX or p*trp*-LUX fusion plasmid grown in MEM-HEPES. Data is depicted as RLU, assays were performed in biological triplicate and error bars represent standard deviation.

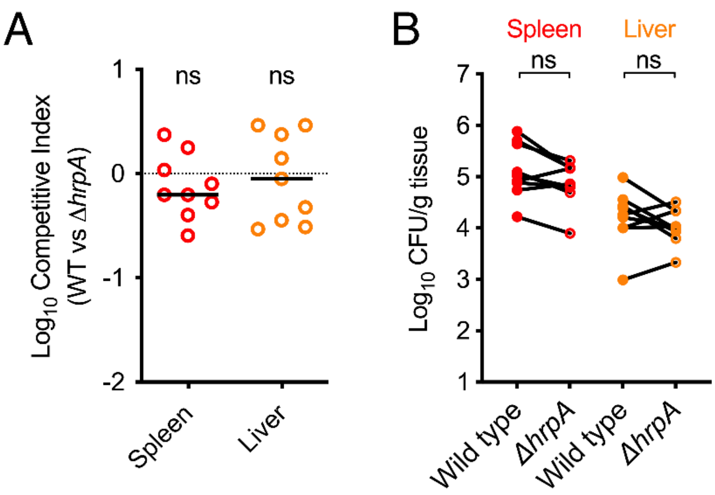

Fig S4. (A) Competitive index of WT UPEC versus Δ*hrpA* during murine BSI. Mice were infected intravenously with a 1:1 mixture of both strains. The data points indicate the fold decrease in Δ*hrpA* CFU recovered per organ in comparison to WT UPEC CFU. (B) The mean CFU per organ of each strain in each animal. ns indicates data not significant as determined by Wilcoxon signed rank or Wilcoxon matched pairs signed rank tests.

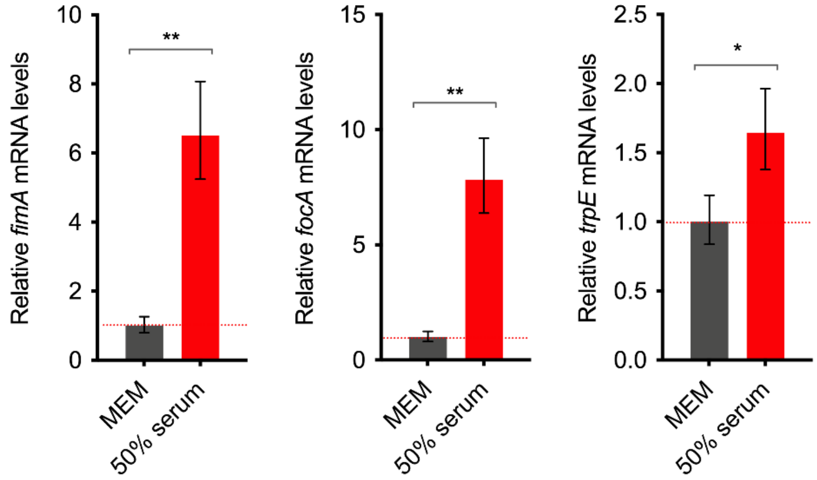

Fig S5. RT-qPCR analysis of relative *fimA, focA* and *trpE* transcript levels in RNA derived from WT UPEC cultured in MEM-HEPES with or without 50% human serum. The red dotted line indicates the WT expression threshold. Data is derived from three biological replicates and error bars indicate standard deviation. * and ** indicate $p < 0.05$ and $p < 0.01$ as determined by Student *t*-test.

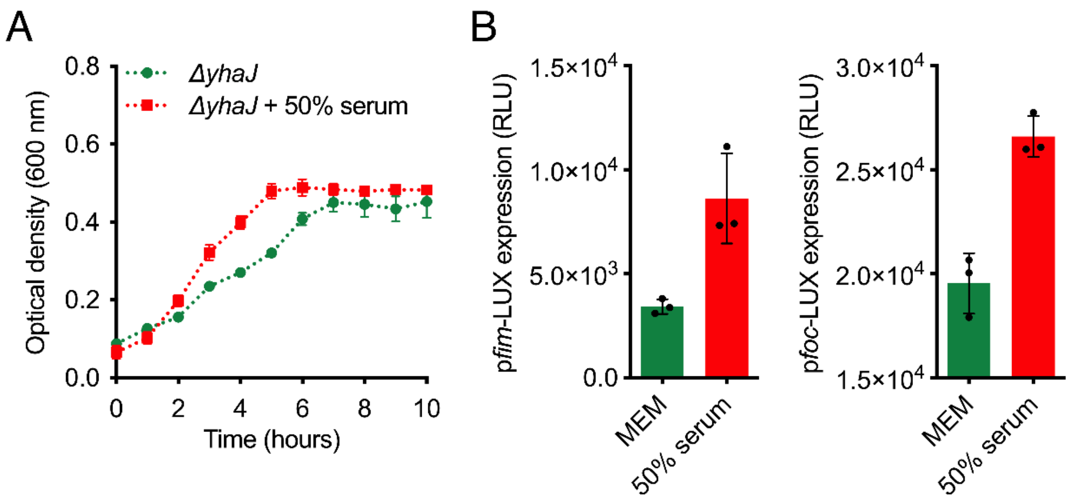

Fig S6. (A) Growth of WT UPEC and Δ*yhaJ* in MEM-HEPES minimal media with and without 50% human serum. (B) Transcriptional reporter assays of Δ*yhaJ* carrying a p*fim*-LUX or p*foc*-LUX fusion plasmid grown in MEM-HEPES with or without human serum. Data is depicted as RLU, assays were performed in biological triplicate and error bars represent standard deviation.

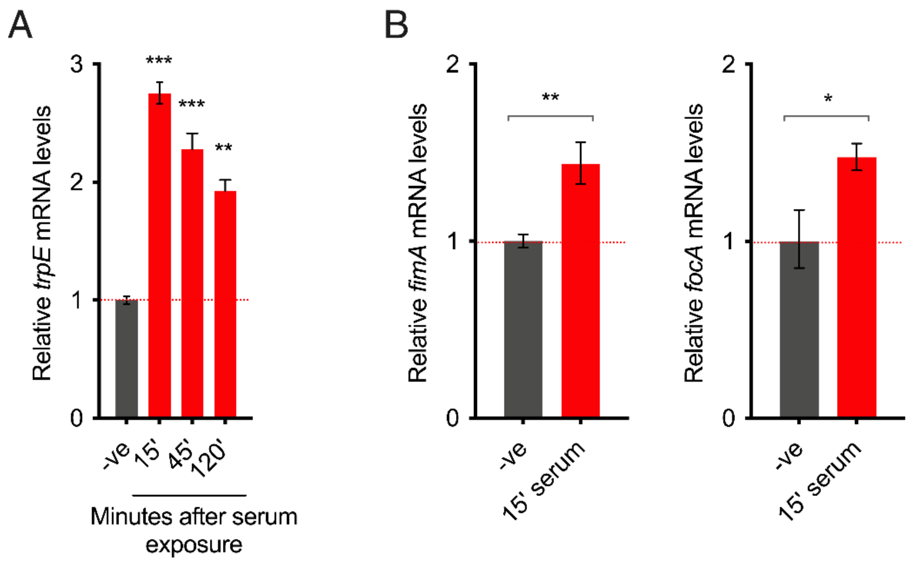

Fig S7. (A) RT-qPCR analysis of relative *trpE* transcript levels in RNA derived from WT UPEC cultured in MEM-HEPES alone or spiked with 50% human serum for the duration indicated. The red dotted line indicates the WT expression threshold. Data is derived from three biological replicates and error bars indicate standard deviation. ** and *** indicate $p < 0.01$ and $p < 0.001$ respectively, as determined by Student *t*-test. (B) RT-qPCR analysis of relative *fimA* and *focA* transcript levels in RNA derived from WT UPEC cultured in MEM-HEPES alone or spiked with 50% human serum for 15 minutes.

Table S1. Bacterial strains used in this study

Strain	Description	Source
CFT073	Wild type uropathogenic *E. coli* isolated from patient with urosepsis	Prof Rodney Welch
ΔyhaJ-kan	CFT073 *yhaJ* deletion mutant (Kan^R)	Connolly *et al., PLoS Path* 2016
ΔyhaJ	CFT073 *yhaJ* clean deletion mutant	Connolly *et al., PLoS Path* 2016
ΔhrpA	CFT073 *hrpA* deletion mutant (Kan^R)	This study
ΔhrpA/yhaJ	CFT073 *hrpA* and *yhaJ* deletion mutant (Kan^R)	This study
Δfoc	CFT073 *focABCDFGHsfaCDY* deletion mutant (Kan^R)	This study
Δtrp	CFT073 *trpLEDCFBA* deletion mutant (Kan^R)	This study
BL21 (DE3)	Commercial *E. coli* overexpression strain	New England Biolabs

Table S2. Plasmids used in this study

Plasmid	Description	Source
pET28-YhaJ	N-terminally 6xHistidine tagged YhaJ cloned into pET28a (Kan^R)	Connolly *et al., PLoS Path* 2016
pSUPROM	Complementation plasmid for constitutive expression of cloned genes under the control of the *tat* promoter (Kan^R)	Jack *et al., EMBO J* 2004
pSU-YhaJ	YhaJ coding sequence from CFT073 cloned into pSUPROM (Kan^R)	This study
pMK1-LUX	Reporter construct for fusing promoters to the *luxCDABE* cassette (Amp^R)	Karavolos *et al., BMC Genomics* 2008
p*fim*-LUX	The *fim* operon promoter region cloned into pSUPROM (Amp^R)	This study
p*foc*-LUX	The *foc* operon promoter region cloned into pSUPROM (Amp^R)	This study
p*trp*-LUX	The *trp* operon promoter region cloned into pSUPROM (Amp^R)	This study
pKD46	Lambda Red recombinase expressing plasmid, permissive at 30°C (Amp^R)	Datsenko and Wanner, *PNAS* 2000
pKD3	Template plasmid for amplification of the FRT-Chl^R-FRT cassette	Datsenko and Wanner, *PNAS* 2000
pKD4	Template plasmid for amplification of the FRT-Kan^R-FRT cassette	Datsenko and Wanner, *PNAS* 2000
pCP20	Plasmid expressing FLP recombinase by thermal induction, permissive at 30°C (Amp^R)	Datsenko and Wanner, *PNAS* 2000

Table S3. Primers used in this study

Primer	Description	Sequence
*foc*_LR_fwd	Forward primer for cassette amplification from pKD3/4 flanked with 50bp directly either of the *foc* locus	CTGATGTAACTTTTATCTGT TTCAGTGAAGCATGCCCACA AACTGAGTTAGTGTAGGCTG GAGCTGCTTC
*foc*_LR_rev	Reverse primer for cassette amplification from pKD3/4 flanked with 50bp directly either of the *foc* locus	TCAGATAAAAGAACATAA ATGCTGGCTGCTGACAAG TCTGTGAAAAAGGACATA TGAATATCCTCCTTAG
*foc*_check_fwd	Forward check primer for amplifying the *foc* locus	GGCCATGCAGTAAAACCG
*foc*_check_rev	Reverse check primer for amplifying the *foc* locus	TACAAGCGCGCATCAGTC
*trp*_LR_fwd	Forward primer for cassette amplification from pKD3/4 flanked with 50bp directly either of the *trp* locus	CATCGAACTAGTTAACTAGT ACGCAAGTTCACGTAAAAAG GGTATCGACAGTGTAGGCTG GAGCTGCTTC
*trp*_LR_rev	Reverse primer for cassette amplification from pKD3/4 flanked with 50bp directly either of the *trp* locus	CTTCGTTAAAGAAAGTTAAA ATGCCGCCAGCGGAACTGGC GGCTGCGGAACATATGAATA TCCTCCTTAG
*trp*_check_fwd	Forward check primer for amplifying the *trp* locus	TGGCTGTGCAGGTCGTAA
*trp*_check_rev	Reverse check primer for amplifying the *trp* locus	TTGACGTTCGACAGGGGT
*hrpA*_LR_fwd	Forward primer for cassette amplification from pKD3/4 flanked with 50bp directly either of the *hrpA* gene	AACAACAAAAATTGACCTTT ACTGCCTTGCAGCAGCGGCT GGATTCGCTGGTGTAGGCTG GAGCTGCTTC
*hrpA*_LR_rev	Reverse primer for cassette amplification	CCGGCGCTAAATGCTTACCG GGTTTTAGATTTATCAGGCA

	from pKD3/4 flanked with 50bp directly either of the *hrpA* gene	AATAGCAGTGCATATGAATA TCCTCCTTAG
*hrpA*_check_fwd	Forward check primer for amplifying the *hrpA* gene	GCATAGAAAGCCGCGATG
*hrpA*_check_rev	Reverse check primer for amplifying the *hrpA* gene	ACTCCGCAAGCCTGACAT
*yhaJ*_pSU_fwd	Forward primer for cloning *yhaJ* into pSU-PROM at *BamH*I	TATAGGATCCATGGCCAA AGAAAGGGCATT
*yhaJ*_pSU_rev	Reverse primer for cloning *yhaJ* into pSU-PROM at *Xba*I	TATATCTAGATTATTTTCCG CTAAAAAG
P*fim*_pLUX_fwd	Forward primer for cloning the *fim* promoter into pMK1-LUX at *EcoR*I	CCCGAATTCTTGCCGGATT ATGGGAAAGA
P*fim*_pLUX_rev	Reverse primer for cloning the *fim* promoter into pMK1-LUX at *BamH*I	CCCGGATCCGGCAGTCGT TCTGTACACTTT
P*foc*_pLUX_fwd	Forward primer for cloning the *foc* promoter into pMK1-LUX at *EcoR*I	CCCGAATTCAAAGGATAA TAAGTGGCTGACTTG
P*foc*_pLUX_rev	Reverse primer for cloning the *foc* promoter into pMK1-LUX at *BamH*I	CCCGGATCCTGCTGTGC CATTCGATACTC
P*trp*_pLUX_fwd	Forward primer for cloning the *trp* promoter into pMK1-LUX at *EcoR*I	CCCGAATTCGCACTCCC GTTCTGGATAAT
P*trp*_pLUX_rev	Reverse primer for cloning the *trp BamH*I promoter into pMK1-LUX at	CCCGGATCCCCTTCGC AGGTTAGCAGTT
*fimA*_qPCR_fwd	*fimA* transcript forward primer	CAATCGTTGTTCTGTCGGC

*fimA*_qPCR_rev	*fimA* transcript reverse primer	AAGTGAACGGTCCCACCA
*focA*_qPCR_fwd	*focA* transcript forward primer	CTGCTGTCACCACGGTTAAT
*focA*_qPCR_rev	*focA* transcript reverse primer	CCGTCTGATCGAATGAGTTAGTG
*trpE*_qPCR_fwd	*trpE* transcript forward primer	CGCAGATATCGACAGCAAAGA
*trpE*_qPCR_rev	*trpE* transcript reverse primer	GTGTCACTTAATGCTGTAATGCG
*gapA*_qPCR_fwd	*gapA* transcript forward primer	CCGTTGAAGTGAAAGACGG
*gapA*_qPCR_rev	*gapA* transcript reverse primer	CCCATTTCAGGTTAGCCG
*foc*_EMSA_fwd	*foc* promoter region forward primer	CGCGAGAAATATAAGCCTGCA
*foc*_EMSA_rev	*foc* promoter region reverse primer	CGCACACCGTATCTGTCATC
*hrpA*_EMSA_fwd	*foc* promoter region forward primer	TCGGATTTGTAGCCAGGTCA
*hrpA*_EMSA_rev	*foc* promoter region reverse primer	TCTGTCATAACGTTAGTGGGC
*trp*_EMSA_fwd	*foc* promoter region forward primer	TCATTAGGCGGGCTGGGTAT
*trp*_EMSA_rev	*foc* promoter region reverse primer	CCATCGGAAGCTGTGGTATG
*deaD*_EMSA_fwd	*foc* promoter region forward primer	GAAAAGTGTGAACCGGCTCA
*deaD*_EMSA_rev	*foc* promoter region reverse primer	CAACGGATAACACCTCGCTC

Table S4. Mass spectrometric parameters for individual metabolites: precursor to fragment ion transition, voltage potential (Q1), collision energy (CE) and voltage potential (Q3).

Compound	RT (min)	Precursor m/z	Product m/z	Q1 Pre Bias (V)	CE	Q3 Pre Bias (V)
Tryptophan	7.4	205.10	188.15	-16.00	-12.00	-23.00

Supplementary references

1. K. A. Datsenko, B. L. Wanner, One-step inactivation of chromosomal genes in Escherichia coli K-12 using PCR products. *Proc. Natl. Acad. Sci. U. S. A.* **97**, 6640–6645 (2000).
2. R. L. Jack, *et al.*, Coordinating assembly and export of complex bacterial proteins. *EMBO J.* **23**, 3962–3972 (2004).
3. M. H. Karavolos, *et al.*, Adrenaline modulates the global transcriptional profile of Salmonella revealing a role in the antimicrobial peptide and oxidative stress resistance responses. *BMC Genomics* **6**, 458 (2008).
4. J. P. R. Connolly, N. O'Boyle, N. C. A. Turner, D. F. Browning, A. J. Roe, Distinct intraspecies virulence mechanisms regulated by a conserved transcription factor. *Proc. Natl. Acad. Sci. U. S. A.* **116**, 19695–19704 (2019).
5. J. P. R. Connolly, N. O'boyle, A. J. Roe, Widespread strain-specific distinctions in chromosomal binding dynamics of a highly conserved escherichia coli transcription factor. *MBio* **11**, 1–6 (2020).
6. M. D. Robinson, D. J. McCarthy, G. K. Smyth, edgeR: A Bioconductor package for differential expression analysis of digital gene expression data. *Bioinformatics* **26**, 139–140 (2009).
7. F. Strino, M. Lappe, Identifying peaks in ChIP-seq data using shape information. *BMC Bioinformatics* **17** (2016).
8. D. Szklarczyk, *et al.*, The STRING database in 2021: Customizable protein-protein networks, and functional characterization of user-uploaded gene/measurement sets. *Nucleic Acids Res.* **49**, D605–D612 (2021).
9. C. S. Hung, K. W. Dodson, S. J. Hultgren, A murine model of urinary tract infection. *Nat. Protoc.* **4**, 1230–1243 (2009).

10. S. N. Smith, E. C. Hagan, M. C. Lane, H. L. T. Mobley, Dissemination and systemic colonization of uropathogenic Escherichia coli in a murine model of bacteremia. *MBio* **1**, e00262-10 (2010).

11. K. J. Livak, T. D. Schmittgen, Analysis of relative gene expression data using real-time quantitative PCR and the 2-ΔΔCT method. *Methods* **25**, 402–408 (2001).

12. J. P. R. Connolly, *et al.*, A Highly Conserved Bacterial D-Serine Uptake System Links Host Metabolism and Virulence. *PLoS Pathog.* **12** (2016).
